# Improving Opportunities for Patients to Raise Concerns During Ward Rounds: A Quality Improvement Project

**DOI:** 10.7759/cureus.113406

**Published:** 2026-07-26

**Authors:** Diya Kothari, Edrich Richards, Yusuf Alghabra, Yasmin Hirsi, Madihah Ali, Imogen Vining, Victoria Metaxa

**Affiliations:** 1 Guy's, King's College, and St Thomas' (GKT) School of Medicine, King's College London, London, GBR; 2 Department of Critical Care Medicine, King's College Hospital NHS Foundation Trust, London, GBR; 3 Department of Critical Care Medicine, King’s College Hospital NHS Foundation Trust, London, GBR

**Keywords:** escalation, mixed-methods, patient concerns, quality improvement study, ward rounds

## Abstract

Introduction

Martha's Rule was introduced following the death of 13-year-old Martha Mills, whose signs of sepsis were overlooked despite concerns raised by her parents. It can often be difficult to voice concerns in a busy clinical setting, but one key opportunity for them to be addressed is the daily ward round.

Aims

This study aimed to increase the proportion of patients and relatives asked about concerns, to increase the proportion of concerns addressed by doctors, and to identify discrete types of concerns raised.

Methods

We observed 15 ward rounds between October 2024 and February 2025 and documented interactions using a premade observational tool. "Intervention 1" provided patients with colour-coded prompt cards assessing their conditions and concerns. "Intervention 2" briefed doctors to ask patients about their concerns. Data was evaluated using both quantitative and qualitative methods. Quantitative data were analysed using run charts to assess trends and variation over time, with median values used as a reference line to identify shifts and patterns. Qualitative responses were categorised and presented using pie charts to illustrate the distribution of themes and proportions.

Results

Pre-intervention observations showed that, though concerns raised were mostly addressed, doctors often failed to explicitly ask about concerns. Intervention 1 was discontinued due to poor patient engagement. Run chart analysis demonstrated improved communication following Intervention 2, with the proportion of patients directly asked about their concerns increasing from 2/8 (25%) to 17/20 (85%) pre-intervention to 9/9 (100%) at its peak. The proportion of concerns addressed during ward rounds remained high throughout and reached 7/7, 9/9, and 8/8 (100%, 100%, and 100%), over three consecutive days post-intervention. Symptom-related concerns were the most common both before and after the intervention, suggesting that the intervention improved the communication of concerns rather than altering their nature. Intervention 2 increased the proportion of patients asked about their concerns but did not affect the proportion of concerns addressed. Common concerns elicited were "pain", "other symptoms", "equipment", and "discharge plans", which remained unchanged post-interventions.

Conclusion

Intervention 2 had the most positive effect in prompting doctors to ask patients about concerns but remains unsustainable. This project highlights the challenges around patient engagement during ward rounds and the need for consistent data collection surrounding interventions. Alternative and sustainable solutions to promote patient participation in their care may need to be considered.

## Introduction

Effective patient-centred communication is an important part of safe and high-quality healthcare. Patients who feel heard and involved in decisions about their care generally report better satisfaction, improved understanding of management plans, and more confidence in the healthcare team [1]. However, effective communication during inpatient ward rounds is often overlooked due to workload pressures, staffing shortages, time constraints, and the hierarchical structure of hospital medicine [2]. Recent studies continue to identify poor communication as one of the main contributors to patient safety incidents and preventable mistakes within healthcare settings [3].

Ward rounds are one of the few regular opportunities for patients to directly communicate concerns with the clinical team. Despite this, a recent study exploring patient experiences of ward rounds has demonstrated that whilst some patients are satisfied with ward rounds, many still feel excluded from discussions surrounding their care, particularly in fast-paced inpatient environments [4]. Patients may feel uncertain about when it is appropriate to speak, may not wish to interrupt clinicians, or may feel overwhelmed by the ward round process itself [4].

Research has shown that a structured ward round template improves patient safety by standardising communication during ward rounds [5]. Communication barriers, including language difficulties, cognitive impairment, and severe illness, can reduce patient engagement, although visual communication tools may help patients express concerns [6]. However, evidence for patient-directed interventions remains mixed, as their success often depends on factors such as staffing pressures, information accessibility, and clinician engagement [7].

The project was inspired by the introduction of Martha's Rule across NHS England. Martha Mills was a 13-year-old girl who died from unrecognised sepsis in 2021 despite repeated concerns raised by her parents. In 2023, a coroner concluded that earlier escalation to intensive care would likely have changed the outcome, leading to the development of Martha's Rule by NHS England [8]. The initiative aimed to improve patient safety by giving patients, relatives, and staff the option to access a rapid 24/7 critical care outreach team and required structured communication with patients during hospital admissions [8]. Recent NHS England data suggested that Martha's Rule may already be improving the escalation of deteriorating patients and supporting patients and relatives feel more empowered to raise concerns [9]. This is because patient involvement is being increasingly recognised as an important part of patient safety and high-quality healthcare. Patients and relatives are often able to identify deterioration, communication gaps, or unmet concerns earlier than healthcare professionals, particularly in acute inpatient settings [9].

We explored the component of Martha's Rule which proposes that patients are asked about their condition, in a structured manner, at least once a day [10]. Our rationale was that patients were not being prompted or given enough opportunity to raise concerns; we recognised that the most regular point of contact for patients with their doctors is the morning ward round and, therefore, prioritised simple, low-cost interventions that would be optimal at such times.

The aim of this quality improvement project was to evaluate whether two communication-focused interventions could improve opportunities for patients to raise concerns during inpatient ward rounds. The primary objectives were to increase the proportion of patients who were directly asked about concerns during ward rounds and the proportion of patient concerns that were addressed by the clinical team. A secondary objective was to identify the most common categories of patient concerns raised during ward rounds.

## Materials and methods

This quality improvement project was undertaken at King's College Hospital (KCH) by five fourth-year medical students under the supervision of an intensive care consultant. The project explored whether simple communication-focused interventions improved opportunities for patients to raise concerns during ward rounds and whether doctors addressed them. The primary outcome measures were the proportion of patients directly asked about concerns during ward rounds and the proportion of patient concerns that were addressed by the clinical team. The secondary outcome measure was the categorisation of the concerns raised by patients. The main problem identified was the lack of structure and awareness surrounding how patients could raise concerns during ward rounds, which may have reduced opportunities for concerns to be identified and addressed.

Baseline data collection took place across two inpatient wards at KCH. Ward rounds led by consultants, registrars, and foundation doctors were observed over several weeks to gain a representative picture of current communication practices. Seven ward rounds were observed during this period. As this was a quality improvement project, no formal sample size calculation was undertaken. The duration of data collection was determined by the project timeline and was designed to capture multiple ward rounds before and after each intervention to allow comparison across the two Plan-Do-Study-Act (PDSA) cycles. A standardised observational form (Appendix A) was used to record whether doctors directly asked patients about concerns, whether patients raised concerns spontaneously, whether concerns appeared to be addressed, and the types of concerns raised.

Prior to data collection, all members of the project team were familiarised with the observational tool and agreed standards for each recorded outcome to promote consistency. Where possible, observations were conducted independently by two members of the project team before reaching a consensus regarding the recorded observations. Any discrepancies were resolved through discussion to minimise observer bias and improve consistency. Decisions regarding whether concerns had been "addressed" were made jointly based on whether clinicians explicitly acknowledged the concern and provided an explanation, reassurance, or clinical action such as a referral or investigation in response. Concerns that were acknowledged but deferred without explanation or action were not considered addressed.

Eligible participants were adult inpatients (aged 18 years or over) who were present during consultant-led ward rounds on the participating wards and were able to take part in routine communication with the clinical team. Consecutive eligible patients observed during the study period were included.

Patients were excluded if they were unavailable during the ward round, were unable to communicate due to a tracheostomy, or lacked capacity without a relative or advocate present to support communication or where observation was considered clinically inappropriate by the treating clinical team, for example, if the patient was acutely unwell or receiving urgent care.

Baseline observations showed that doctors directly asked patients about concerns in approximately 52/84 (62%) of encounters. Not all concerns raised by patients appeared to be addressed during the ward round. Several possible contributing factors were identified. These included limited awareness of escalation pathways, staffing pressures, communication barriers, limited interpreter availability, and differences in communication styles based on the level of training of resident doctors.

**Figure 1:**
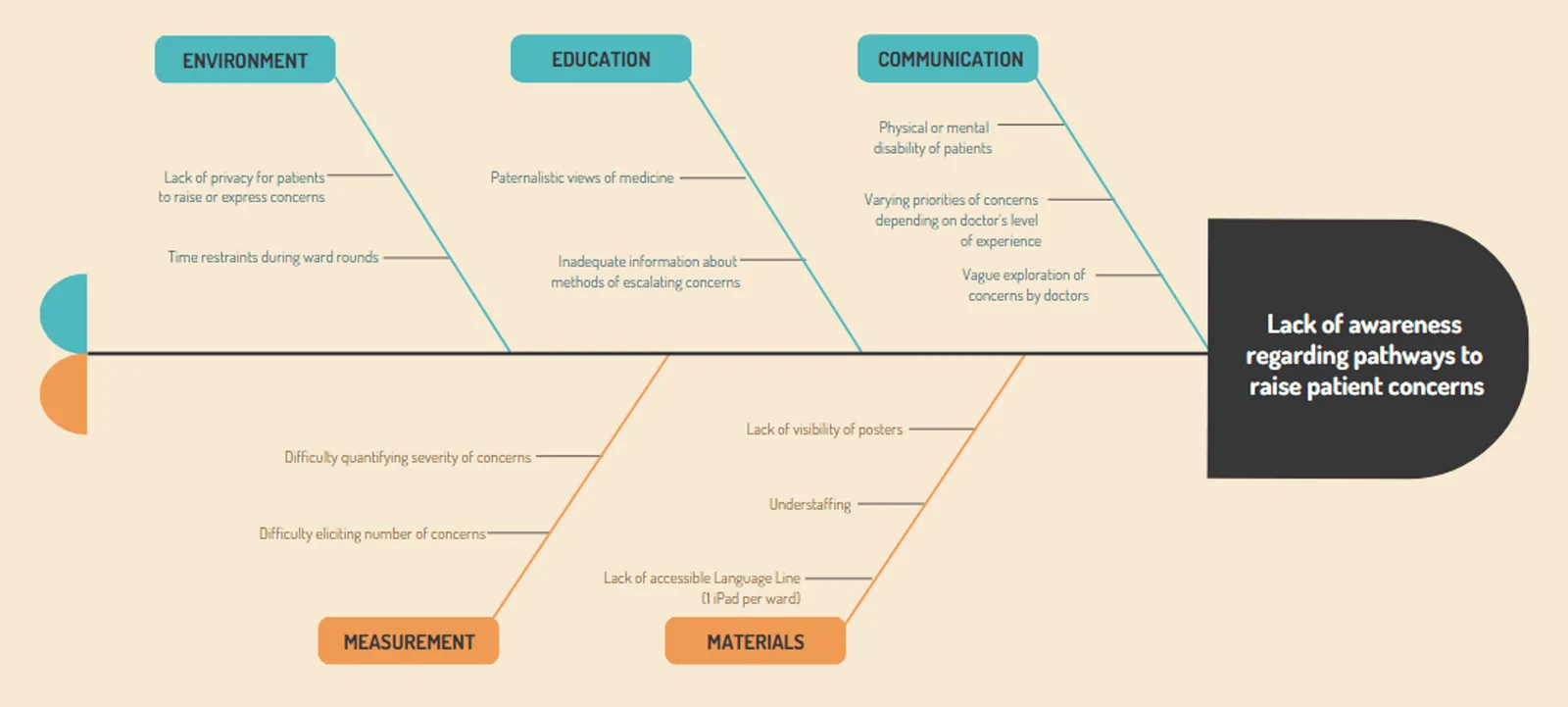
Fishbone diagram Fishbone diagram illustrating the factors contributing to the lack of awareness regarding pathways to raise patient concerns.

These factors are summarised in the fishbone diagram (Figure 1).

Interventions

Since understaffing issues and the busy ward environment were beyond the scope of the project, the interventions focused on improving communication and awareness around patient concerns. We undertook two full PDSA cycles in total. The interventions were designed to provide prompts for both patients and clinicians. The first intervention aimed to empower patients to communicate concerns, while the second aimed to prompt clinicians to consistently encourage patients to raise concerns during ward rounds.

PDSA Cycle 1

During the observation phase, we noted that only 38/76 (50%) of patients raised concerns unprompted, so the first intervention aimed to provide patients with another way of expressing concerns while also prompting doctors to ask about them during ward rounds.

Deciding to incorporate a visual medium to elicit patient concerns, we constructed a laminated traffic light prompt card to be given to patients and their families (Appendix B). The sheets were laminated so they could be reused and to minimise unnecessary waste. We hoped that by providing a visual prompt, more concerns could be raised and subsequently addressed by doctors.

Data collection during this phase relied on digitally collected patient surveys completed over three separate days. Members of the project team also briefed the doctors and nurses in charge about the intervention and handed out the cards with erasable markers prior to the ward rounds.

PDSA Cycle 2

The second intervention focused on doctors rather than patients to improve communication on ward rounds. Members of the project team attended handover meetings before ward rounds and explained the purpose of the project to doctors. Doctors were briefed to actively ask patients whether they had any concerns or questions during ward rounds. Data was collected with the same observational form as baseline data collection to allow an easier comparison with the previous data.

Ethical considerations

This quality improvement study was done in accordance with the UK Health Research Authority (HRA) guidelines. The HRA research criteria determine this study as a service evaluation; therefore, the NHS Research Ethics Committee (REC) was not required. This project did not require ethics committee approval or institutional review board (IRB) review, as participation from patients and doctors was voluntary and anonymous and no incentives were offered.

The authors declare no conflicts of interest with the project.

We used the Standards for QUality Improvement Reporting Excellence (SQUIRE) reporting guideline [11] to draft this manuscript and the SQUIRE 2 reporting checklist when editing, included in Appendix C.

## Results

We created run charts [12] from our quantitative data to track changes over time. The median was calculated using baseline data and used as a reference line to assess changes following each intervention. Run charts were interpreted according to established quality improvement rules by assessing for shifts (six or more consecutive data points above or below the median), trends (five or more consecutive points continuously increasing or decreasing), the number of runs (crossings of the median), and any astronomical data points (values that were obviously different from the remaining observations). These rules were used to determine whether observed changes were likely to represent true improvement rather than random variation. Qualitative data was analysed using pie charts to visually represent any changes.

**Figure 2:**
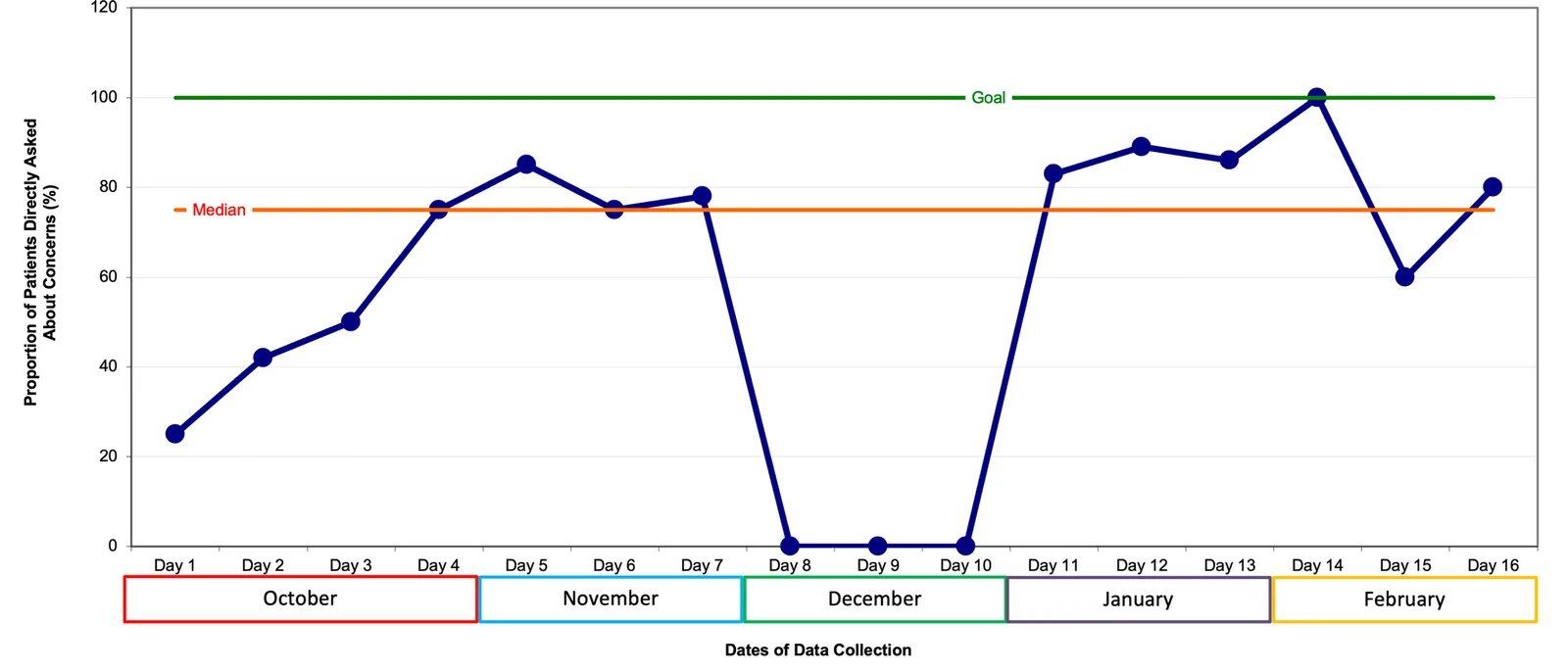
Run chart showing the proportion of patients who were directly asked about concerns by doctors during the ward round between October 2024 and February 2025. Interventions for both PDSA cycles are labelled PDSA: Plan-Do-Study-Act

Figure 2 demonstrates that before the intervention, the percentage of patients who were directly asked about their concerns by doctors during ward rounds ranged from 2/8 (25%) to 17/20 (85%), demonstrating variability in practice. The run chart demonstrates no shifts, one upward trend near the beginning, no astronomical data point, and a total of six runs. This indicates that there was an improvement due to Intervention 2 and less likely due to chance. During Intervention 1, data collection was limited as ward rounds were not shadowed and we relied on patients to participate and complete the online survey. Consequently, no patients were recorded as being asked about their concerns. Following Intervention 2, there was a notable improvement, with the proportion of patients directly asked about concerns rising to 6/7 (86%) and maintaining this run until a post-intervention peak of 9/9 (100%) on day 14. This metric fluctuated about the median after this point but remained higher, on average, than both the pre-interventional and first cycle results.

**Figure 3:**
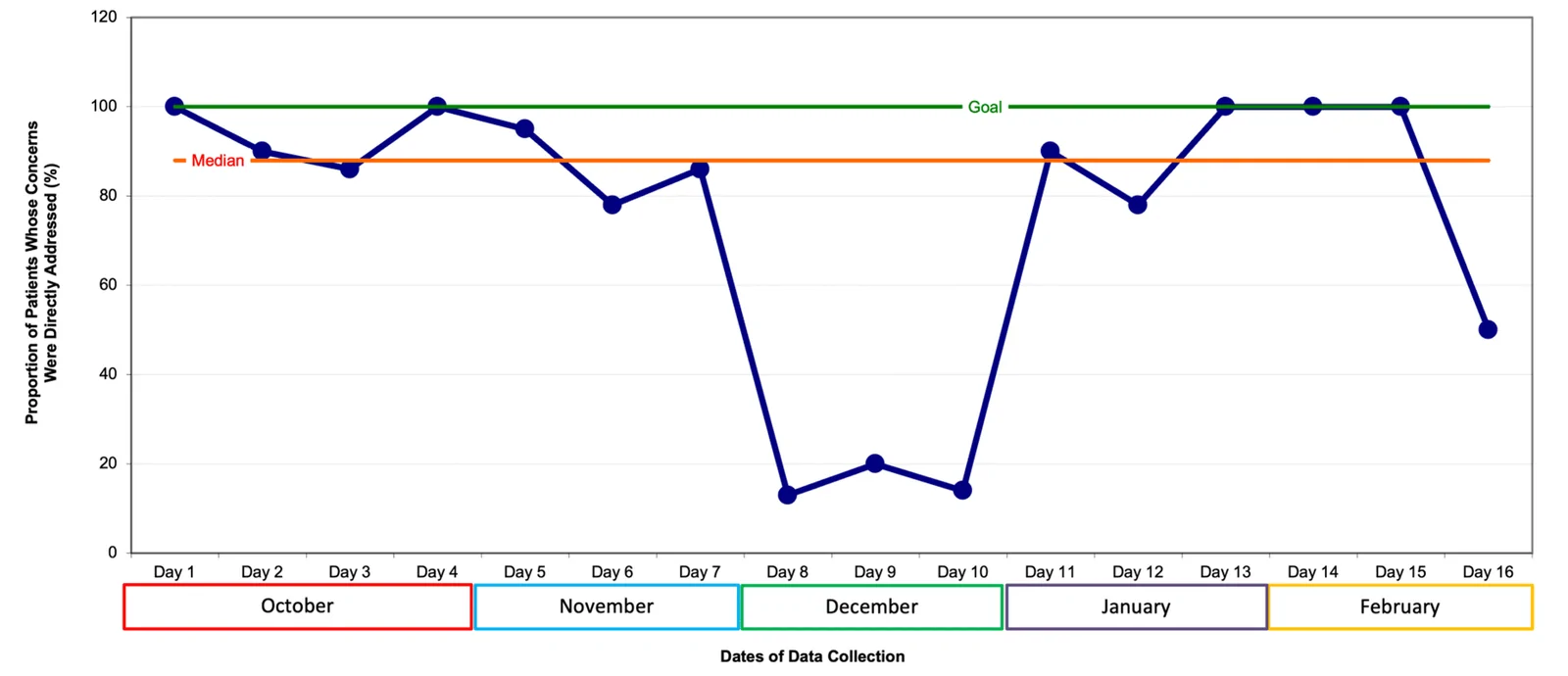
Run chart showing the proportion of patients whose concerns were directly addressed by doctors during the ward round between October 2024 and February 2025. Interventions for both PDSA cycles are labelled PDSA: Plan-Do-Study-Act

Pre-intervention data in Figure 3 showed that 10/13 (77%) to 8/8 (100%) of patient concerns were addressed during ward rounds. The run chart showed no shifts, trends, or astronomical data points, with six runs, an appropriate number for 15 data points. This suggests the observed changes are unlikely due to chance, indicating a probable improvement. In line with the previous trend for the first intervention, on day 8, the proportion of concerns addressed dropped to 13% and maintained this run until day 10. With Intervention 2, there were similar levels to pre-intervention data, with values ranging from 2/4 (50%) to 9/9 (100%). Notably, there was an immediate increase in concerns addressed to 7/8 (88%) on day 11, with a post-intervention peak run of 7/7, 9/9, and 8/8 (100%, 100%, and 100%) for three days. Though day 16 exhibited a decline in concerns addressed to 2/4 (50%), the overall trend was positive in this metric, suggesting a sustained improvement.

**Figure 4:**
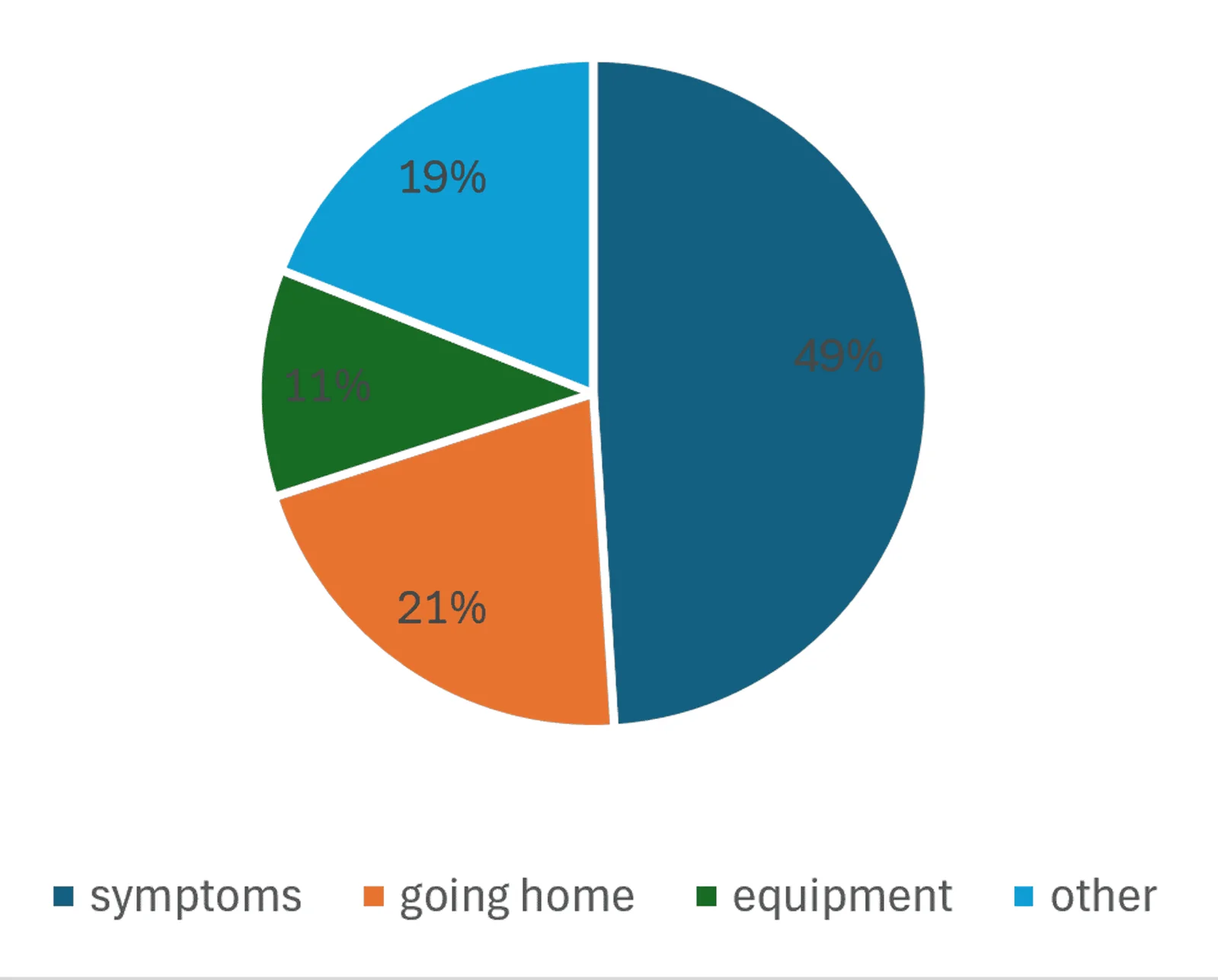
Pre-intervention themes of concerns

**Figure 5:**
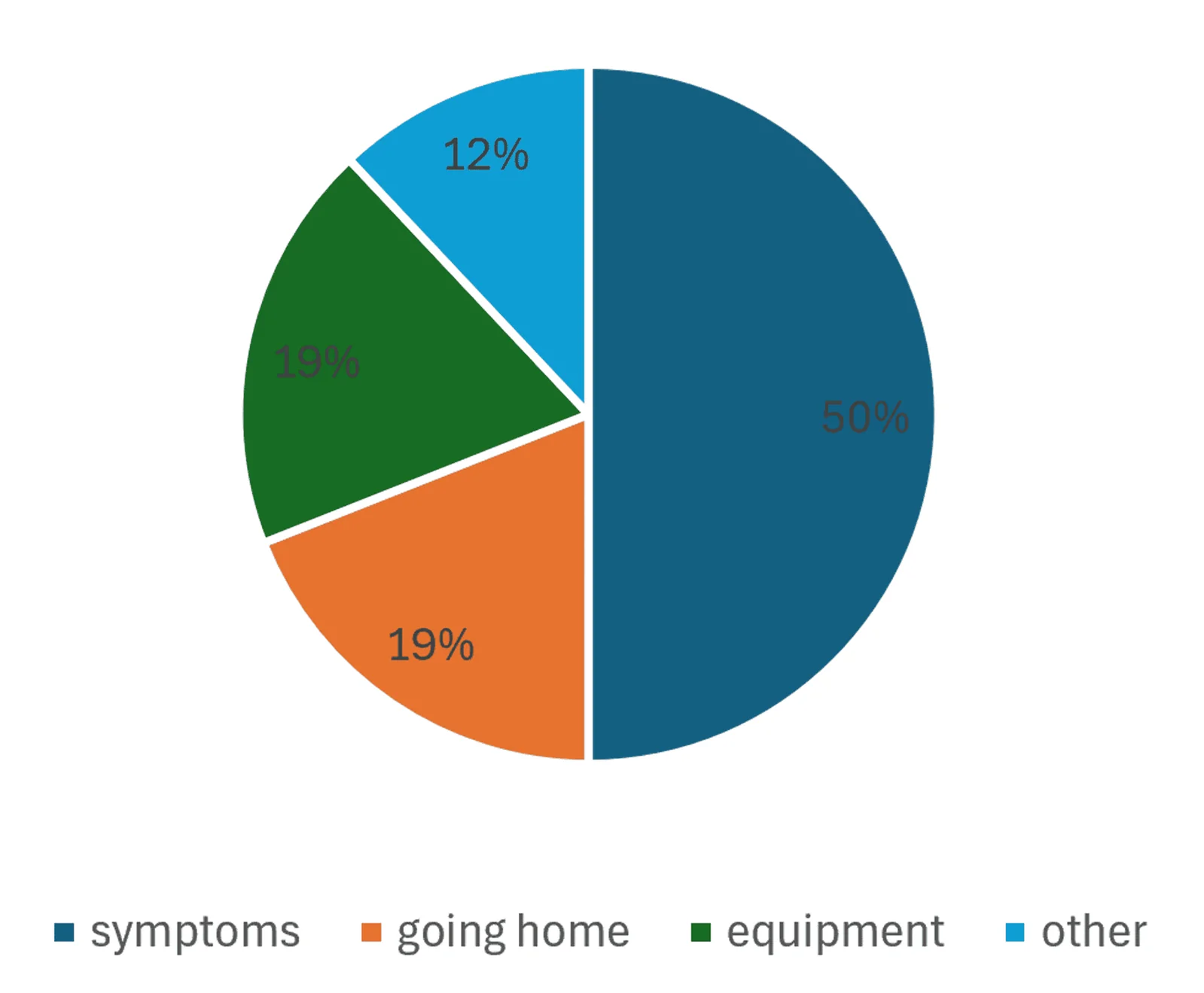
Post-intervention themes of concerns

We categorised the most common concerns into "symptoms", "going home", "equipment", and "other" (Figure 4 and Figure 5). "Symptoms" were most often related to pain or breathlessness, "going home" encompassed concerns about discharge, and "equipment" refers to issues with comfort or compliance of machinery at the bedside. "Other" concerns raised were primarily legal or familial care inquiries. Symptoms experienced remained the most common patient concern, both pre- and post-intervention. Figure 4 and Figure 5 are very similar, indicating that the interventions mostly influenced the communication of concerns rather than the nature of them.

## Discussion

The results indicate that the second intervention was more effective at improving the number of patients asked about concerns but did not significantly affect the proportion of concerns addressed. This suggests that although the intervention improved opportunities for patients to raise concerns, it did not clearly change whether concerns were addressed. Since only 38/76 (50%) patients initially raised concerns unprompted, this intervention may have made doctors become aware of a greater number of patient concerns overall, even if the proportion of them addressed remained similar. One possible explanation for this is that doctors were still working within the same workload pressure and time constraints during ward rounds. A recent systematic review demonstrated that structured communication tools may improve patient engagement and communication quality during ward rounds, although measurable improvements in patient outcomes are often harder to demonstrate consistently [13]. Improving communication alone may therefore be insufficient to significantly improve patient outcomes.

Although the first intervention did not significantly improve communication overall, it appeared to provide an unexpected benefit in helping patients and relatives overcome language barriers. Some patients and their families appeared more comfortable writing concerns down visually when verbal communication was difficult. Similar findings were reported by D'Souza et al., who demonstrated that visual communication tools may improve communication for patients with limited English [6]. This type of intervention may therefore be particularly useful within multilingual inpatient settings or intensive care environments where communication barriers are more common, helping to reduce healthcare inequality [14].

Overall, these findings suggest that relatively simple interventions can improve opportunities for patients to participate in ward round discussions, although improving the quality of patient-centred communication is likely to require broader cultural change and more complex interventions. Embedding prompts for patient concerns into routine ward round practice may represent a practical step towards strengthening patient engagement while supporting more patient-centred care [5].

Limitations

The first intervention had several limitations. The apparent decline in concerns elicited and addressed following the first intervention implies an entirely detrimental effect of the prompt card; however, given the low likelihood of this in practice, we believe that our data collection method may have negatively skewed the data. In hindsight, the card should have supplemented the ward round observations, rather than being used as the sole method of data collection. Although the concept of using alternative communication was well-received by the healthcare team, many patients struggled to use the prompt card due to their frailty. This suggests that patient-only interventions may be less effective without broader clinician engagement, aligning with Newman et al.'s review, which found limited evidence of their effectiveness [7].

The second intervention was more effective overall in prompting doctors to ask patients about their concerns. However, it depended heavily on active involvement from the project team, including attending handover meetings and the direct shadowing of ward rounds. This raises concerns regarding long-term sustainability. It is also possible that doctors have altered their behaviour due to awareness of being observed (Hawthorne effect) [15]. Briefing doctors immediately before observing ward rounds may have influenced their approach during ward rounds, temporarily improving communication rather than producing a sustainable long-term change. Interventions that rely on repeated prompting are difficult to maintain long-term within busy clinical environments. Embedding prompts into ward round templates or electronic documentation systems may therefore represent a more practical and sustainable solution [5].

This project was conducted across two inpatient wards with a relatively small sample size, limiting the generalisability of the findings. This project also did not formally assess patient satisfaction, clinician perceptions, or the total number of concerns raised before and after the interventions. Including qualitative feedback and patient-reported outcome measures in future studies would provide a better understanding of whether the interventions made meaningful improvements in patient outcomes and safety. Finally, the relatively short follow-up period limited our ability to assess whether the improvements in communication were sustained over time. Future studies should include longer-term follow-up to determine the sustainability of these interventions.

Another study could involve assessing differences in communication across different levels of doctors in training to identify whether this affects the proportion of concerns addressed. Also, extending the intervention to other healthcare staff may improve patient-centred communication across the whole multidisciplinary team as this improves patient care [16]. Improving inclusion of the patient voice likely requires wider cultural change and more complex interventions. We did not expect that small changes alone would fully address these challenges; however, this project aimed to build on previous work through simple interventions that could still have a meaningful impact on patient communication and engagement during ward rounds.

## Conclusions

This quality improvement project highlights the importance and the challenges around patient engagement during ward rounds and the need for consistent data collection surrounding interventions. The second intervention increased the proportion of patients directly asked about concerns, suggesting that relatively simple prompts may positively influence communication behaviours within clinical teams. However, this did not majorly increase the number of concerns being addressed, highlighting many limitations during a busy ward round. The project also demonstrated that patient-only interventions may have limited effectiveness without broader clinician engagement with the intervention. Sustainability remains a major challenge, particularly when interventions rely on active involvement from project teams.

These findings should be interpreted within the context of the study design, as this project focused on improving communication practices during ward rounds rather than measuring changes in patient outcomes. Future work should therefore explore integrating structured communication prompts into routine ward round documentation and electronic systems so that opportunities for patients to raise concerns become embedded within standard clinical practice. Involving patient satisfaction and qualitative feedback may also help determine whether these interventions improve both patient experience and patient safety outcomes.

## Figures and Tables

**Figure 6:**
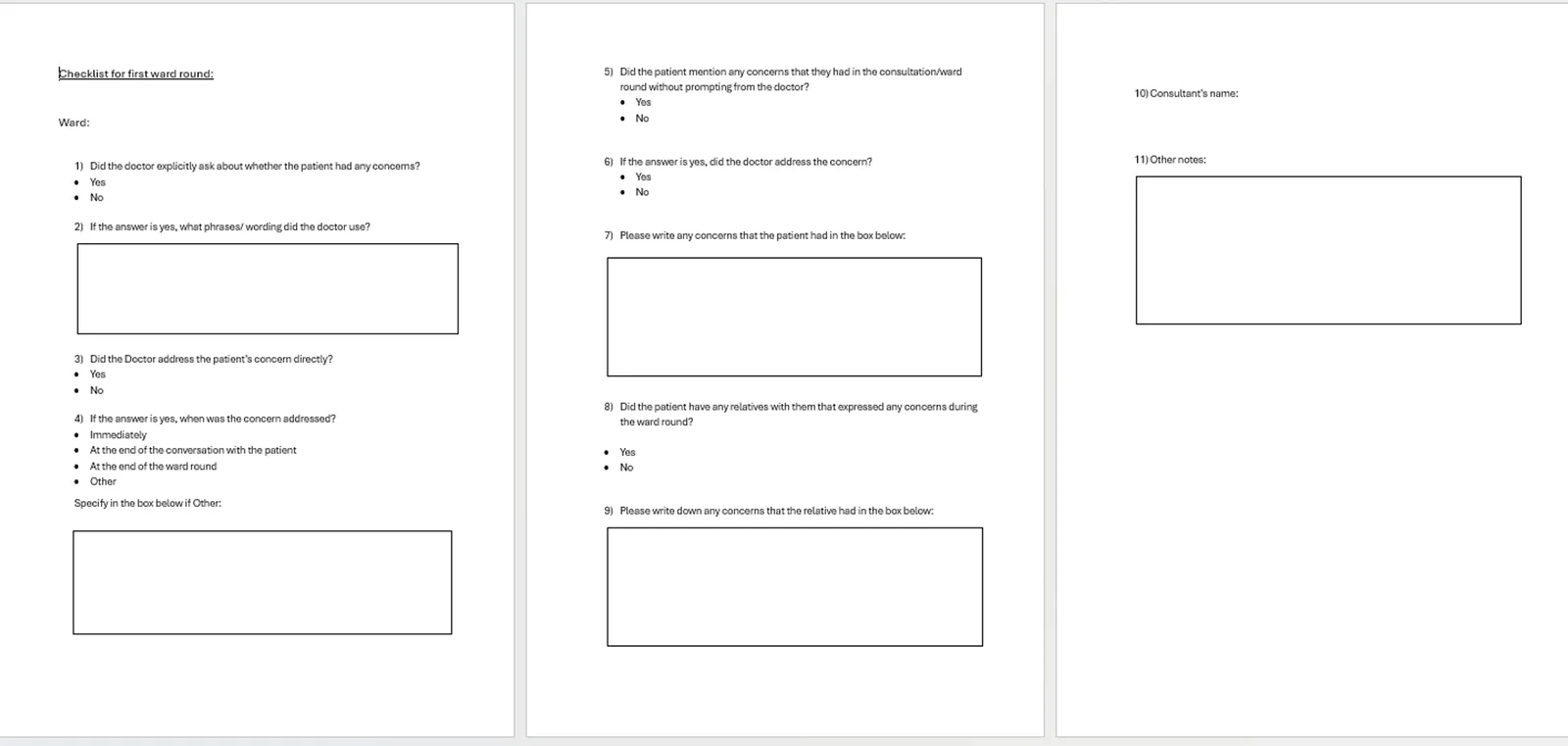
A standardised observational form developed for the project, used to record data during ward rounds

**Figure 7:**
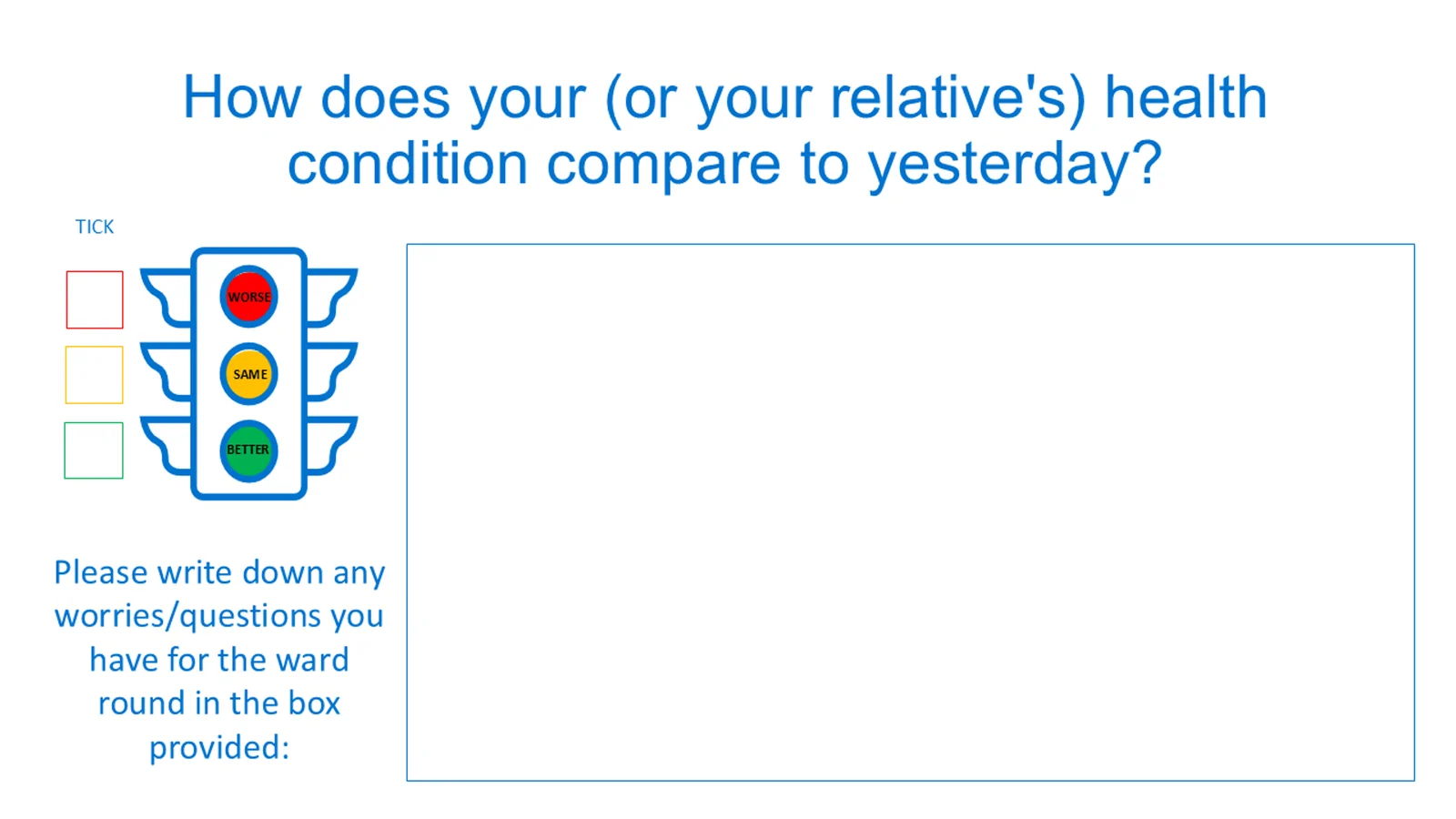
Laminated prompt sheets: Intervention 1

**Figure 8:**
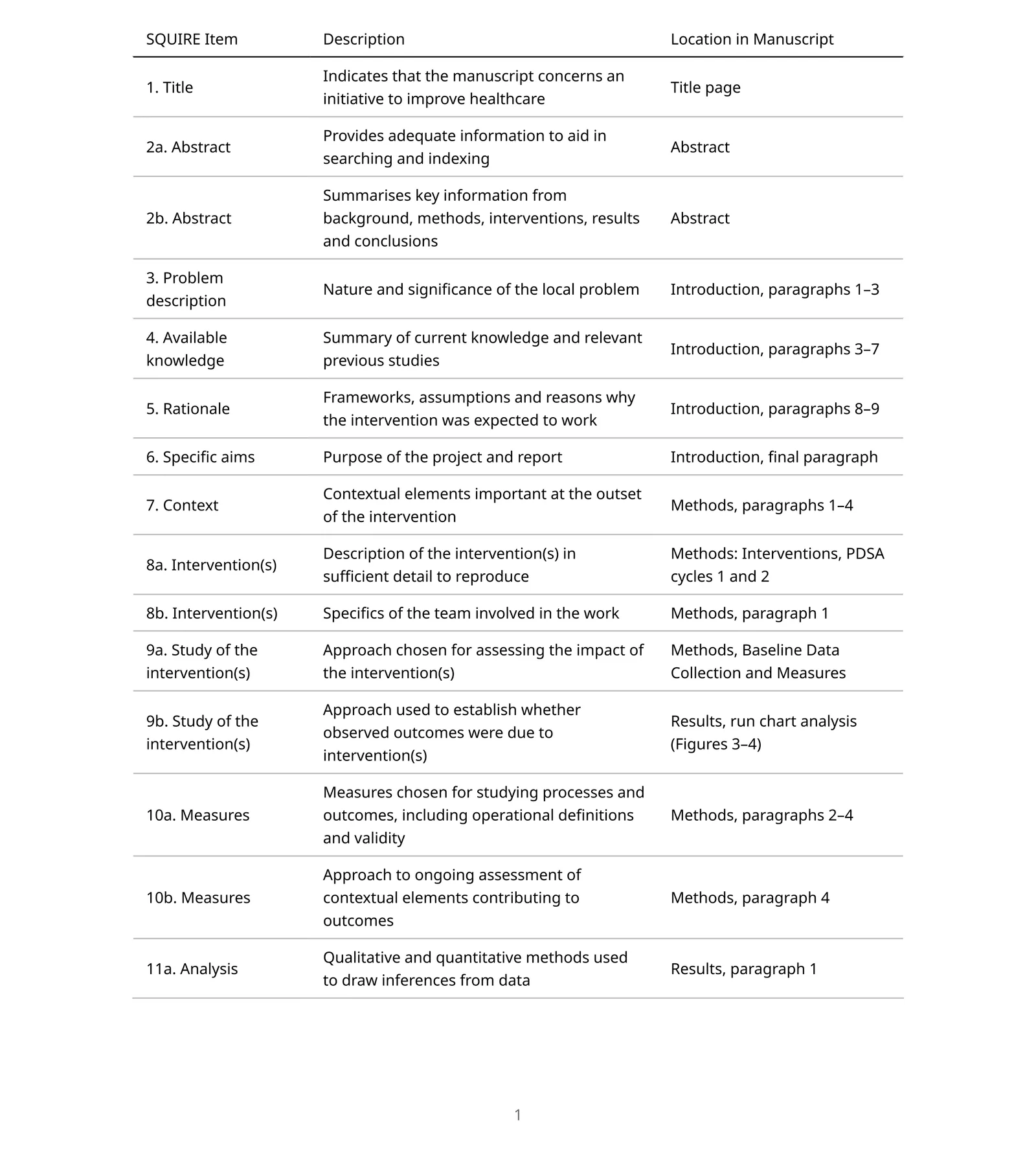
SQUIRE 2 checklist SQUIRE: Standards for QUality Improvement Reporting Excellence Reference: [17]

**Figure 9:**
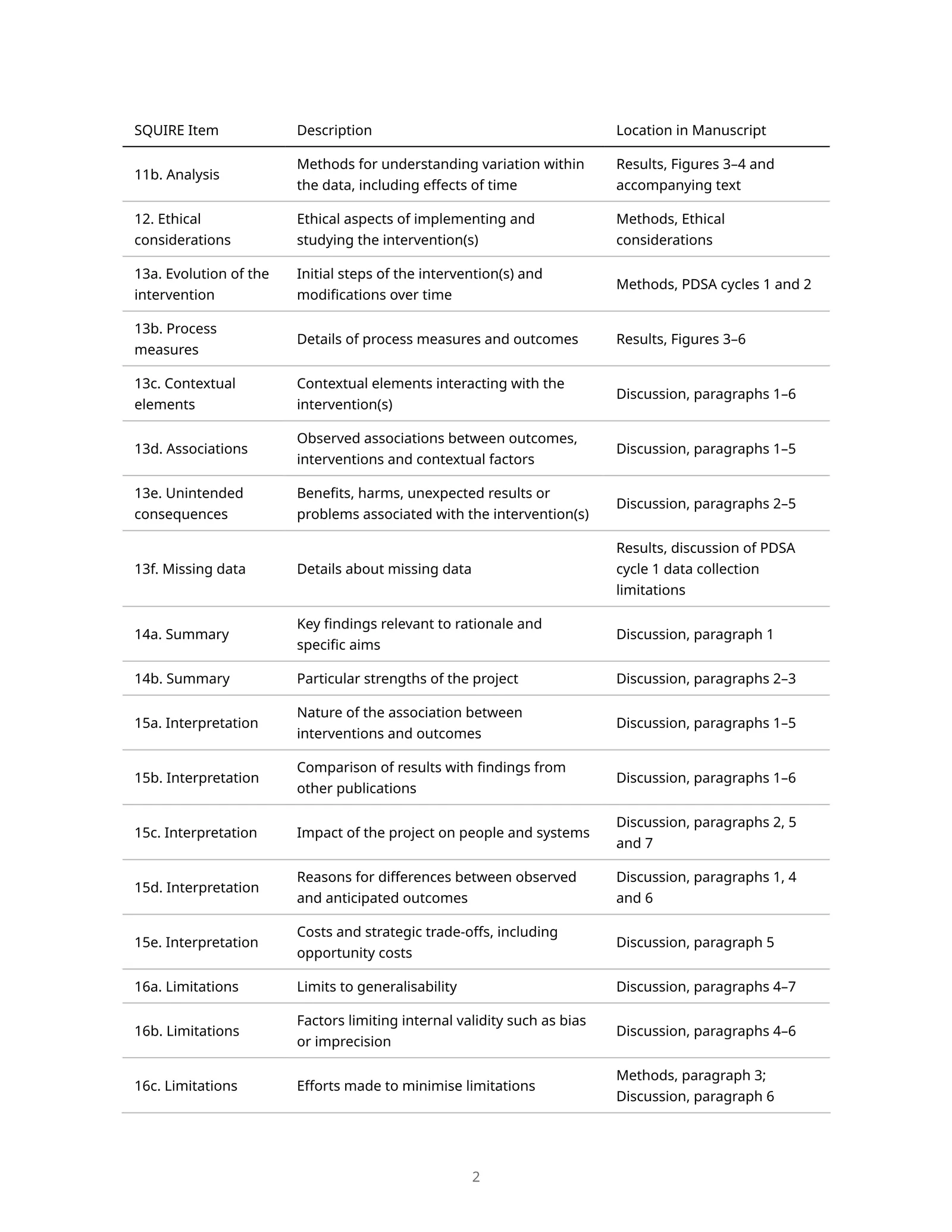
SQUIRE 2 checklist (cont.) SQUIRE: Standards for QUality Improvement Reporting Excellence Reference: [17]

**Figure 10:**
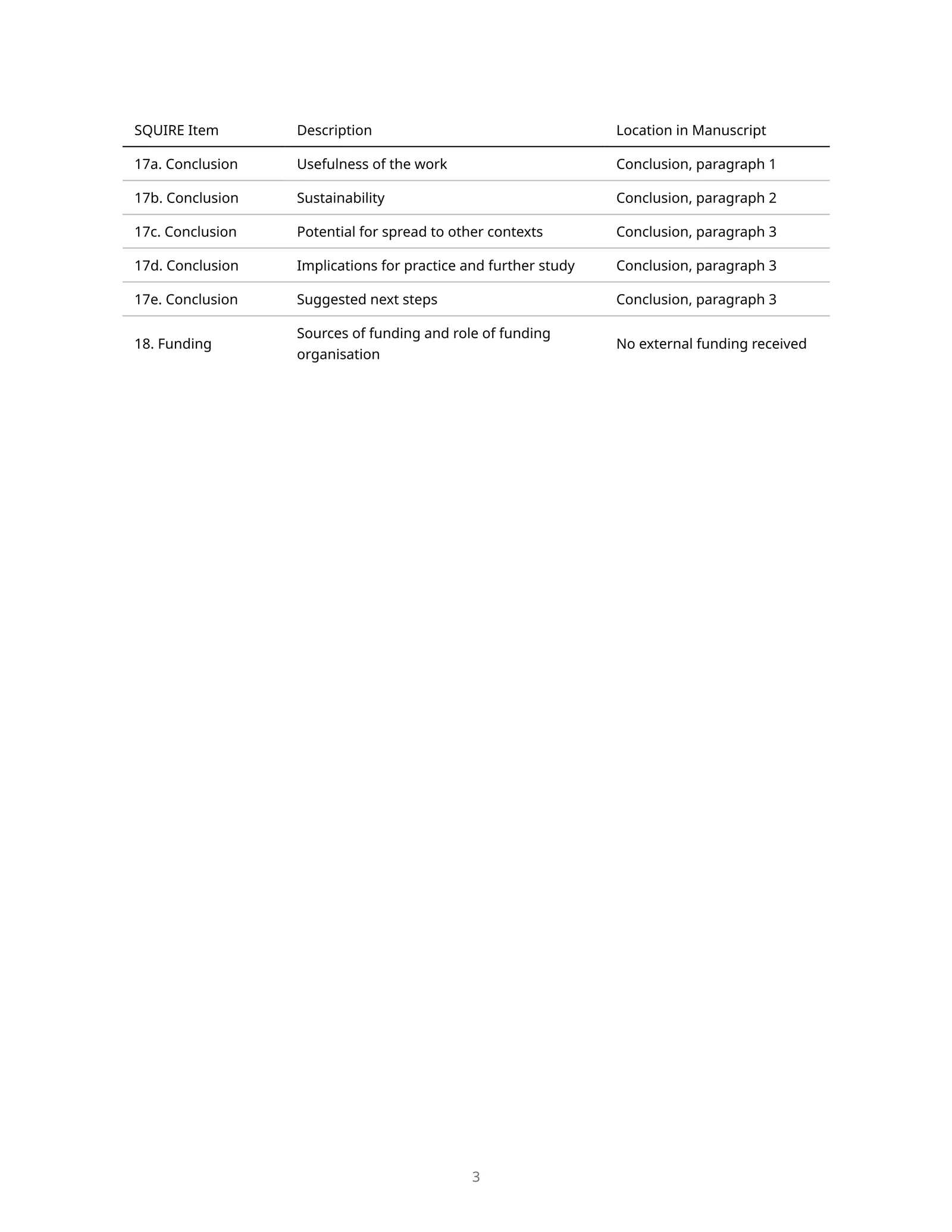
SQUIRE 2 checklist (cont.) SQUIRE: Standards for QUality Improvement Reporting Excellence Reference: [17]

## Appendices

Appendix A

Appendix B

Appendix C
